# Effective Hamiltonians from Spin-Adapted Configuration Interaction

**DOI:** 10.1021/acs.jctc.4c01380

**Published:** 2025-01-08

**Authors:** Arta A. Safari, Nikolay A. Bogdanov

**Affiliations:** Max-Planck-Institute for Solid State Research, 70569 Stuttgart, Germany

## Abstract

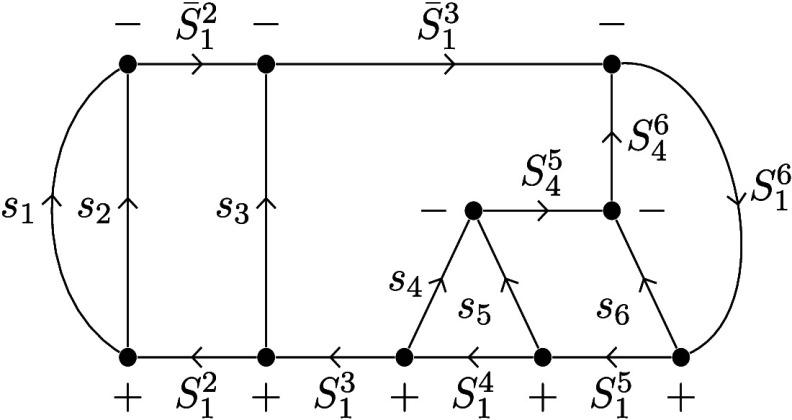

A generalized extraction procedure for magnetic interactions using effective Hamiltonians is presented that is applicable to systems with more than two sites featuring local spins *S*_*i*_ ≥ 1. To this end, closed, nonrecursive expressions pertaining to chains of arbitrary equal spins are derived with the graphical method of angular momentum. The method is illustrated by extracting magnetic couplings from ab initio calculations on a [CaMn_3_^(IV)^O_4_] cubane. An extension to nonsequential coupling schemes proves conducive to expressing additional symmetries of certain spin Hamiltonians.

## 1 Introduction

Transition-metal compounds are a common motif in nature, with examples ranging from single-molecule magnetism^1−7^ to enzymatic catalysis^8−11^ and superconductivity.^12−15^ Their thermally accessible states are typically characterized by localized spins that can be rationalized in terms of phenomenological spin models like the Heisenberg–Dirac–Van Vleck Hamiltonian. If the model admits analytical solutions, energy differences can be fitted to data from experiment or calculation in order to obtain effective interaction constants. While a correspondence of the respective ab initio states to the model can be inferred through evaluation of the orbital-resolved spin correlation function^16,17^ or point group symmetry,^18^ an exact map may not be established, because of the intrinsic difference between ab initio and model spins.^19−21^ A more rigorous method is the effective Hamiltonian approach that renormalizes the full Hamiltonian onto the magnetic subspace.^21,22^ In this procedure, extraneous wave function components are eliminated, which enables an exact map to the model.

To obtain the effective Hamiltonian, hereinafter also called numerical matrix, eigenvectors of the full Hamiltonian are projected onto the model space and must be expressed in a compatible basis. Different choices are possible; for instance, a *S_i_* =  Heisenberg model in the uncoupled basis corresponds to a wave function in Slater determinants. Blocks of interest may then be targeted by choosing a total spin projection *m*_*S*_ common to all considered states. Alternatively, if blocking by total spin *S*_tot_ is desired, the transformation to the coupled basis is given by Clebsch–Gordan coefficients.

Theories formulated in a basis of total spin eigenfunctions,^23−26^ are well-suited to the description of magnetic interactions, since variational degrees of freedom related to recoupling of angular momenta can be treated separately from ones due to mixing of spatial configurations. The resulting compression over spin multiplets has proven particularly beneficial for sparse configuration interaction (CI) solvers like the Density Matrix Renormalization Group (DMRG)^16,27,28^ or Graphical Unitary Group Approach Full CI Quantum Monte Carlo (GUGA–FCIQMC).^29^ Many successful applications to systems consisting of more than two sites with large magnetic moment have already been reported;^17,30−33^ however, to the best of our knowledge, the effective Hamiltonian approach has not yet been formulated in this context. Instead, analysis proceeded via local spin expectation values or DMRG entanglement diagrams.^17,31,32^ The purpose of this letter is threefold: (i) to explain the problem faced in the construction of effective Hamiltonians when more than two sites with *S*_*i*_ ≥ 1 are considered, (ii) to demonstrate how recoupling transformations can be used to resolve this issue, deriving closed expressions for an arbitrary number of equal spins and (iii) to highlight the increased sparsity offered by nonstandard representations of the symmetric or unitary group.

Alternatively, it is possible to work with fragmentation methods^34−37^ that expand the full wave function in a product basis of states with well-defined subsystem quantum numbers. The resulting solutions already contain components that correspond to the uncoupled basis of spin models, making the construction of effective Hamiltonians straightforward, as briefly discussed for the localized active space method^38^ in the Supporting Information.

## 2 Conversion between Coupling Schemes

When two arbitrary magnetic moments on sites A and B are coupled, the resulting states are uniquely characterized by their total spin *S*_tot_, e.g.,  yields *S*_AB_ = {0 ⊕ 1 ⊕ 2 ⊕ 3}. For a two-site model, the Heisenberg Hamiltonian is diagonal in the coupled basis, allowing the derivation of interaction constants from energy differences using Landé’s interval rule. When another spin *S*_C_ is added, the reduction of the tensor product space is generally no longer multiplicity free, i.e., multiple states can have the same *S*_tot_. For example, adding *S*_C_ =  to the case above results in
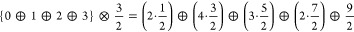
1To resolve this multiplicity problem, states are labeled with two quantum numbers that specify their genealogy, here |*S*_tot_, *S*_AB_⟩. The model matrix is blocked by *S*_tot_, but not guaranteed to be diagonal, unless *S*_AB_ is a good quantum number.

**Figure 1:**
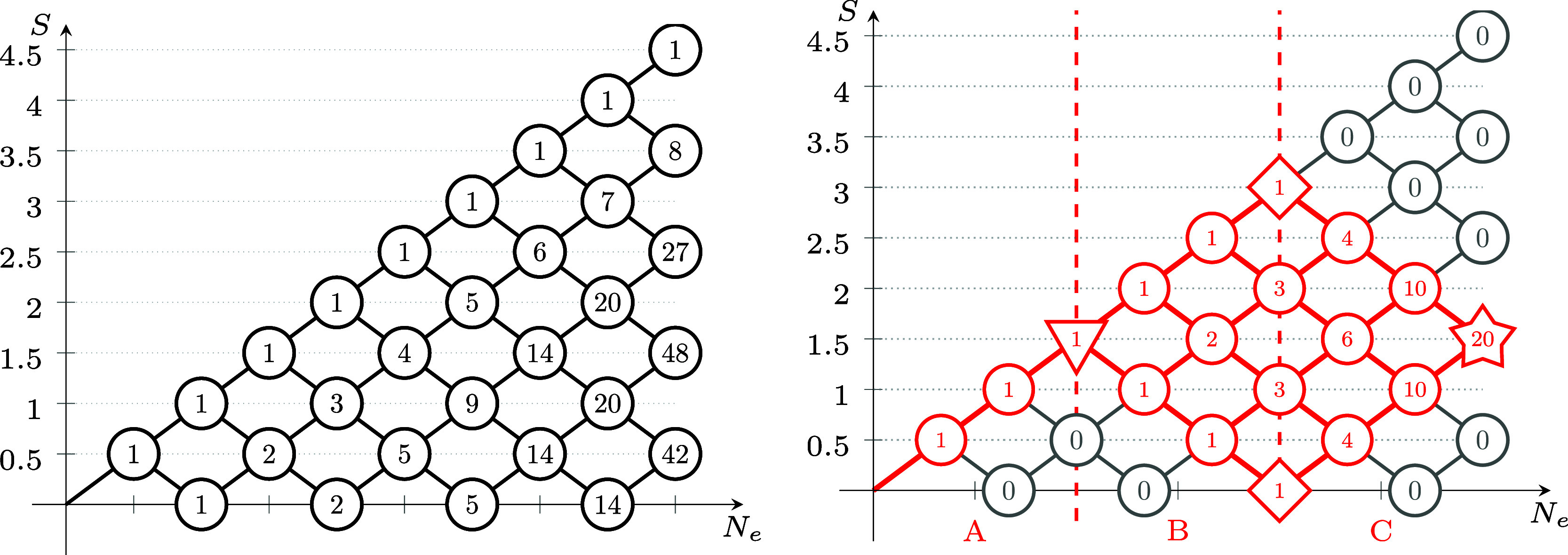
(Left) Genealogical branching diagram in standard form for nine electrons. (Right) Localizing and site-ordering the orbitals expresses Hund’s rule on the first site, marked by a triangle and reduces the number of possible paths. Shown in red are the remaining nonvanishing CSFs with *S*_tot_ = .

Although solutions to the Heisenberg model are characterized by both local spin quantum numbers *S*_A_, *S*_B_, *S*_C_, and *S*_tot_, it is more common in quantum chemistry to couple individual electrons, *s_i_* = , into a cumulative spin up to particle *j*, denoted *S*_*i*_^*j*^. The resulting CSFs correspond to standard representations of the symmetric or unitary group:
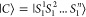
2In the left side of Figure 1, the genealogical graph resembling this coupling scheme is shown for nine electrons in nine spatial orbitals.

It was shown that, for the magnetic system described by Heisenberg-like models, further sparsity may be introduced into the graph, by taking advantage of the stabilization arising from parallel alignment of site spins due to Hund’s rule.^39^ Localizing and site-ordering the orbitals makes the first three electrons correspond to site A, marked by a triangle in Figure 1; energetically penalized non-Hund CSFs with *S*_A_ ≠  get effectively separated. This sparsity manifests in the Full CI expansion as well, by reducing the number of CSFs with appreciable weight.^39^ It bears mentioning, however, that Hund’s rule on sites B and C cannot be expressed with a standard representation in most cases, since local spin quantum numbers corresponding to B and C are not defined in this basis. Collinear states with *S*_AB_ = {0, 3}, marked by squares in Figure 1, are exempt from this problem, since they can only follow from the coupling of two equal spins.^1^

To mimic the local spin operators of the model, electrons on each site should be precoupled before combining them into a total spin:
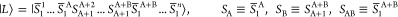
3Bars are used to distinguish quantum numbers occurring in both the standard and nonstandard representations.

|*C*⟩ and |*L*⟩ constitute different coupling schemes that are related by a recoupling transformation:
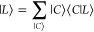
4which we express with the graphical method of spin algebra^40−42^ in the style of Wormer and Paldus.^43^ As an aid to the reader, used ingredients of the graphical method are briefly recapitulated in Appendix A; a comprehensive introduction can be found in ref (43). The diagrammatic approach is not new to quantum chemistry and has already been applied to the study of Serber spin functions,^44−47^ the optimal segmentation of two-electron operators in the GUGA^48^ or subduction coefficients for a general system partitioning *U*(*n* = *n*_1_ + *n*_2_) ⊃ *U*(*n*_1_) × *U*(*n*_2_),^49,50^ among others. Compared to previous work,^49,50^ our derivation of the recoupling expressions is entirely inductive and does not rely on recursion. Although less general, this approach leads to simple closed formulas for systems with equal spins and we expect them to be convenient for studying other model Hamiltonians as well. Following the introductory example, the recoupling of two sites with three electrons into a local spin-adapted representation, *S*(6) ⊃ *S*(3) × *S*(3), will serve as the base case, before treating the general two-site problem *S*(2*n*) ⊃ *S*(*n*) × *S*(*n*) and the chain . Afterward, we illustrate with [CaMn_3_^(IV)^O_4_] and [Fe_4_^(III)^S_4_] cubanes how to use these expressions in practice.

Before ⟨*C*|*L*⟩ is amenable to graphical manipulation, it must be converted to a generalized Clebsch–Gordan coefficient. Inserting the resolution of the identity over all uncoupled states into eq 4, we obtain
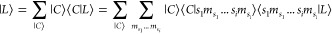
5or graphically for *S*(6) ⊃ *S*(3) × *S*(3):
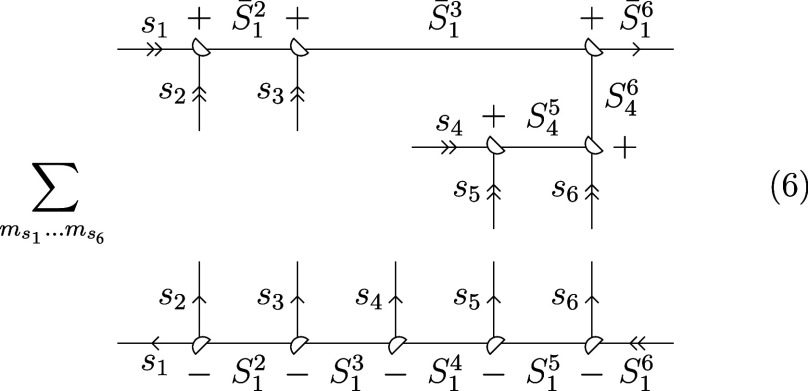
6Contracting over all magnetic quantum numbers and transitioning into 3–*jm* symbols yields
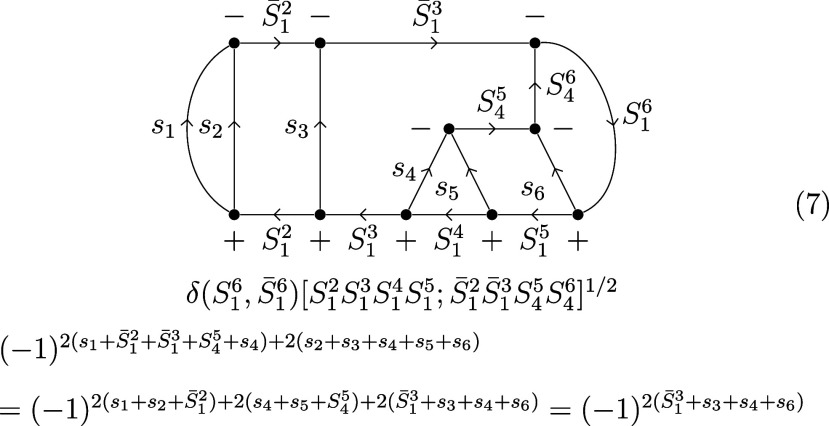
7where the notation [*S*_1_*S*_2_...] = (2*S*_1_ + 1)(2*S*_2_ + 1)... was introduced. To simplify the phase, we make use of the fact that two times the sum of angular momenta connected to the same vertex is an even number. After a two-line separation over *S*_1_^3^/ *S̅*_1_^3^, the arrow directions on *S̅*_1_^2^ and *S*_4_^5^ are reversed:
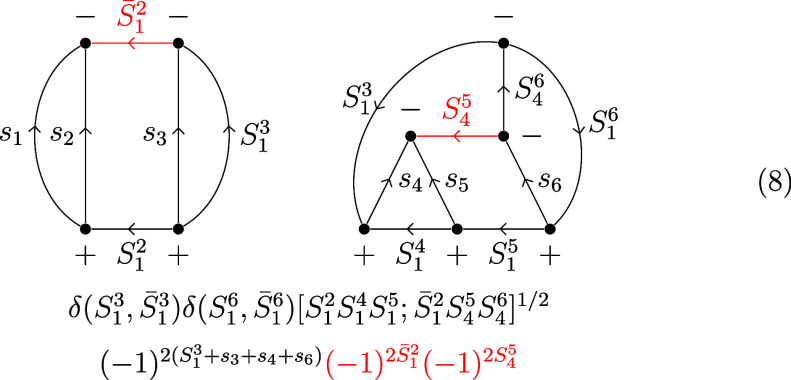
8Following another two-line separation over *S*_1_^2^/*S̅*_1_^2^ and a three-line separation over *S*_4_^5^/*S*_1_^3^/*S*_1_^5^, the 6*j* symbols are brought into standard form, by changing the vertices and arrows marked in red:
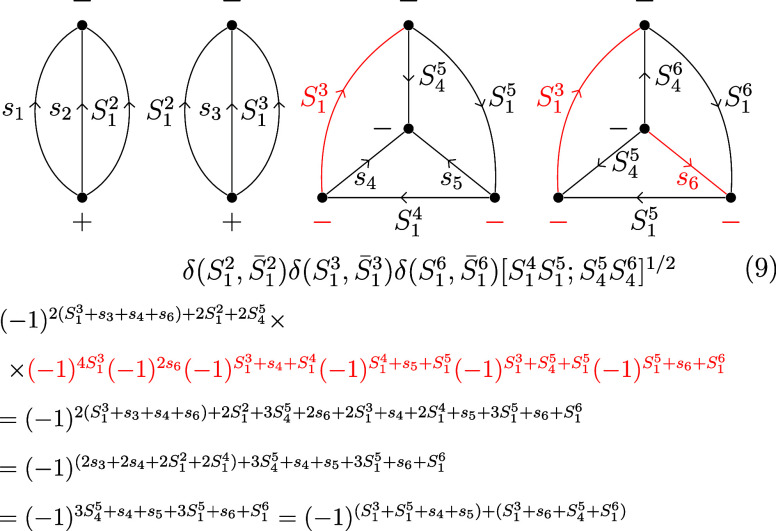
9We also used twice an identity for momenta connected to the same vertex, (−1)^2*S*_12_+2*S*_3_^ = (−1)^2*S*_13_^. The genealogical CSFs |*C*⟩ fulfill the triangle condition for cumulative spins by construction and the 3*j* symbols take the value of 1 accordingly. Substituting *s_i_* = , using the symmetry of the 6*j* symbol under the column permutation (123) ↔ (321) and flipping columns one and three, the recoupling coefficients ⟨*C*|*L*⟩ for *S*(6) ⊃ *S*(3) × *S*(3) take the following form:
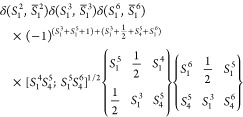
10If we introduce the weighted symbol *W̃* related to the original Racah coefficient *W̅*:^51^
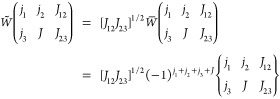
11we can write eq 10 compactly as
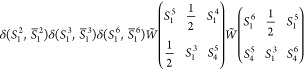
12Equation 12 may be generalized to the case of *k* sites with *S_i_* = . Due to the local spin coupling scheme, different sites remain separable over two lines, entirely similar to *S*_1_^3^/*S̅*_1_^3^ in eq 10 and introduce a factor analogous to eq 11 with a constant offset:
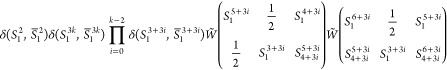
13To generalize to arbitrary spins *S*_*n*_, it is instructive to first observe the action of adding one electron to each of two sites, as in *S*(8) ⊃ *S*(4) × *S*(4) (phases and factors omitted):
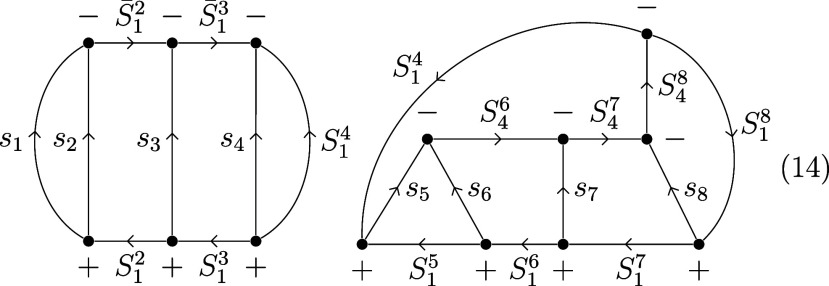
14Compared to eq 8, an additional 3*j* symbol can be factored out from the left drawing and two extra vertices are formed on the right. After separating over three lines twice, *S*_1_^4^/*S*_4_^6^/*S*_1_^6^ and *S*_1_^4^/*S*_4_^7^/*S*_1_^7^, the right-hand side reduces to a product of three 6*j* symbols, i.e., every additional electron contributes one 6*j* coefficient per site. Taking the phases into account as previously, the recoupling coefficient for *S*(2*n*) ⊃ *S*(*n*) × *S*(*n*) becomes a product of *W̃* over site electrons *n*:
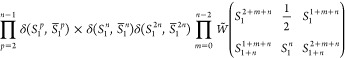
15The general expression for recoupling a chain of equivalent spins *S*_*i*_ ≥ 1 into  is a combination of eqs 13 and 15. Every site adds another 2*n* vertices to the graph, yielding (*n* – 1) 6*j* symbols. In total, the recoupling coefficient is a product of (*k* – 1)(*n* – 1) Racah coefficients with the corresponding Kronecker deltas:
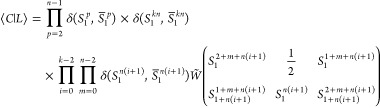
16

## 3 Application: [CaMn_3_^(IV)^O_4_] Cubane

**Figure 2:**
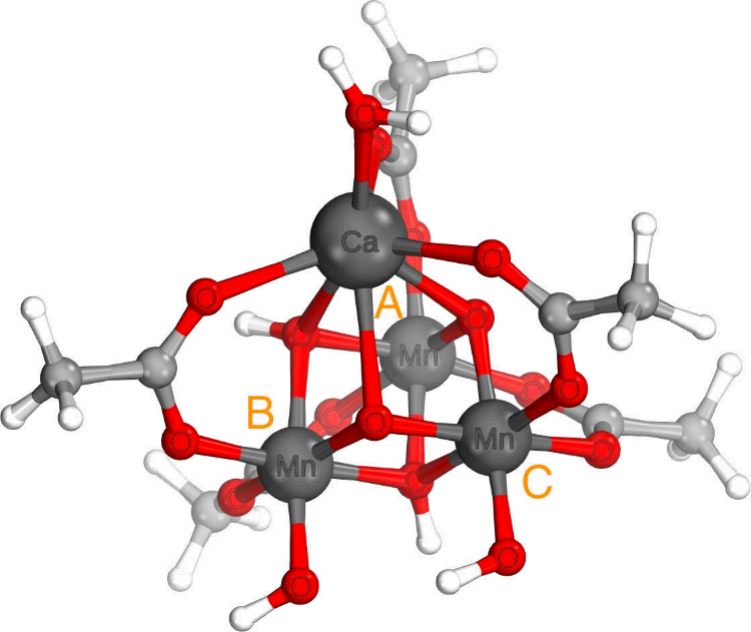
Geometry of the doubly protonated [CaMn_3_^(IV)^O_4_] cubane model.^53^ Magnetic sites are labeled A, B, and C following eq 17. The picture was created with IboView.^54,55^

The [CaMn_3_^(IV)^O_4_] cubane shown in Figure 2 is inspired by a synthetic model of the oxygen-evolving complex of photosystem II. Compared to the experimental structure,^52^ the ligands were simplified and the bridging oxygen in the Mn_A_/Mn_B_ plane protonated,^53^ to study the impact of oxo-protonation on the electronic structure.

In the original study on this compound,^53^ broken-symmetry (BS) DFT calculations, assuming an isotropic, three-parameter Heisenberg model,
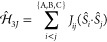
17predicted an octet ground state with couplings constants *J*_AB_ = 6.2 cm^–1^, *J*_AC_ = −40.2 cm^–1^, and *J*_BC_ = −25.4 cm^–1^. Experimental estimates for this structure are not available. With three distinct couplings, eq 17 is no longer analytically solvable, making it an interesting target for the effective Hamiltonian approach. Another advantage of the scheme is a straightforward incorporation of higher-order interactions. In the following, we consider a more general expression including biquadratic interactions *K*_*ij*_(*Ŝ*_*i*_ · *Ŝ*_*j*_)^2^, denoted  (also see section S3 in the Supporting Information).

Apart from the extra recoupling step described in the previous section, formation of the effective Hamiltonian follows the standard prescription,^21,22,56^ i.e., (i) obtain the CI vector in the standard CSF basis; (ii) transform relevant CSFs into the required coupling scheme; (iii) project onto the model space, ensuring that the discarded norm is small; (iv) orthonormalize the projections; and (v) invert the spectral decomposition with the ab initio energies. Magnetic couplings can then be obtained from a mean-square fit of the numerical to the analytical matrix. In the first step, the phase convention of the CI vector should be taken into account. Recoupling CSFs in the unitary group formalism, as, for instance, used in OpenMolcas,^57^ requires a phase to be applied beforehand:^58,59^
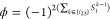
18where {*l*_23_} denotes level indices with values 2 or 3 (negative coupling or doubly occupied) in UGA **d** vector notation.^58^

**Table 1:** Analytical Matrix Corresponding to  in the Basis a

		
		
		
		
	0	

		
		0
		
		
		

a We made the substitutions *J*_3*m*_ = *J*_13_ – *J*_23_, *J*_3*p*_ = *J*_13_ + *J*_23_ and analogously for *K*_3*m*_ and *K*_3*p*_, to make clear that setting *J*_13_ = *J*_23_ and *K*_*ij*_ = 0 diagonalizes the model.^60,61^.

Consistent with the introductory example, the *S*_tot_ =  block of the model matrix is shown in Table 1. The remaining blocks are given in the Supporting Information. Corresponding numerical matrices were derived from calculations performed with OpenMolcas,^57^ using the ANO–RCC basis set^62^ with contractions Ca(20s16p)/[5s4p], Mn(21s15p10d)/[5s4p2d], O(14s9p)/[3s2p], C(14s9p)/[2s1p], H(8s)/[1s] and the Cholesky decomposition of two-electron integrals with a threshold of 10^–4^.^63,64^

A minimal description of the magnetic interactions is achieved by correlating the nine electrons occupying the *t*_2*g*_ orbitals in a CAS(9,9). Spin models usually assume a common spatial component, hence state-averaged CASSCF over the ground state of every multiplicity in eq 1 with equal weight was performed.^2^ The *S*_tot_ =  block of the numerical matrix is shown in Table 2; all other blocks can be found in the Supporting Information.

**Table 2:** Effective Hamiltonian Obtained from CASSCF(9,9) with Labels  Relative to a

				
	147	17		
	17	165	12	
		12	199	7
			7	251

a Values are given in units of cm^–1^. Zeros were omitted to enhance legibility.

Interaction constants are all ferromagnetic at this level of theory and sufficiently different to warrant a 3*J* model, as listed at the end of this section. Due to a mean-field description of virtual ligand-to-metal charge transfer, minimal CASSCF magnetic orbitals tend to be too localized on the metals.^65^ Rather than trying to capture this interaction perturbatively, we expand the variational space within the restricted active space (RAS) framework to keep exact diagonalization feasible.^66^ Six unprotonated bridging O 2p and three σ_Mn–O_ were added to RAS1 and six empty Mn *e*_g_ orbitals to RAS3, with up to two holes and particles, respectively, which we abbreviate as RAS(27,24). Orbital pictures are provided in the Supporting Information. These active orbitals are expected to make the largest differential contributions to the superexchange mechanism and prevent some of the Mn *e*_g_ to be rotated out in favor of O 2p′ orbitals. Analogous to the smaller CAS(9,9), a state-averaged RASSCF over the ground state of each spin sector was performed. Correlating the ligands introduces delocalization tails into the metal-centered orbitals and reduces the retained wave function norm after projection onto the model space to ∼78%. This number may appear small, but a reduced weight of the magnetic manifold is to be expected with an increasing ligand-to-metal charge transfer in the extended active space. The RAS retains a dectet ground state, but compresses the gaps between states, as can be seen for the quartet block in Table 3. All remaining spin sectors are given in the Supporting Information.

**Table 3:** Effective Hamiltonian Obtained from RASSCF(27, 24) with Labels , Relative to a

				
	79	40		
	40	89	28	
		28	107	16
			16	136

a Values are given in units of cm^–1^. Zeros were omitted to enhance legibility.

In contrast to the minimal CAS, the coupling constants from RASSCF, listed in the third row of Table 4, indicate an almost symmetric interaction between Mn_A_/Mn_C_ and Mn_B_/Mn_C_. Biquadratic contributions remain negligible, supporting the simplified model employed in ref (53).

**Table 4:** Magnetic Coupling Constants (in Units of cm^–1^) Derived from CASSCF(9,9) and Different Fully Translated Pair Density Functionals with a RASSCF(27,24) Reference Wave Function, as well as the BS–DFT Estimate from ref (53)^a^

	*J*_12_	*J*_13_	*J*_23_	*K*_12_	*K*_13_	*K*_23_	*b*	*R*^2^
BS-DFT^53^	6.2	–40.2	–25.4	–	–	–	–	–
CASSCF(9,9)	–6.6	–27.7	–20.0	0.0	0.0	0.0	–0.01	1.0000
RASSCF(27,24)	–3.5	–22.2	–3.7	0.0	0.0	–0.1	–0.16	0.9999
+MCPDFT(ftPBE)	19.8	5.8	19.8	–0.1	0.3	–0.3	–0.14	0.9998
+MCPDFT(ftBLYP)	21.9	7.9	21.9	–0.1	0.3	–0.3	–0.14	0.9998
+MCPDFT(ftSCAN-E0)	54.0	23.4	53.9	–0.3	0.5	–0.6	–0.21	0.9998

a The final two columns show the intercept of the mean square fit (in units of cm^–1^) and the coefficient of determination.

With an improved reference wave function, it makes sense to also consider dynamic correlation effects outside the active space. Here, we settled for Multiconfigurational Pair Density Functional Theory (MCPDFT),^67−69^ due to its favorable cost-to-performance ratio compared to RASPT2.^70^ Notably, the tested functionals consistently predict a quartet ground state with purely antiferromagnetic couplings while maintaining symmetric *J*_12_/*J*_23_ exchange pathways, as shown in the last three rows of Table 4. Couplings obtained with the SCAN-E0^71^ functional are more than twice as large as those from PBE^72,73^ or BLYP,^74−76^ but display the same qualitative trend. Considering excitation level restrictions of the RASSCF and basis set limitations, our estimates are unlikely to be converged. Full CASSCF calculations are required to understand the discrepancy between the BS–DFT and RASSCF+MCPDFT results, which will be the subject of future work.

## 4 Nonsequential Coupling Patterns

**Figure 3:**
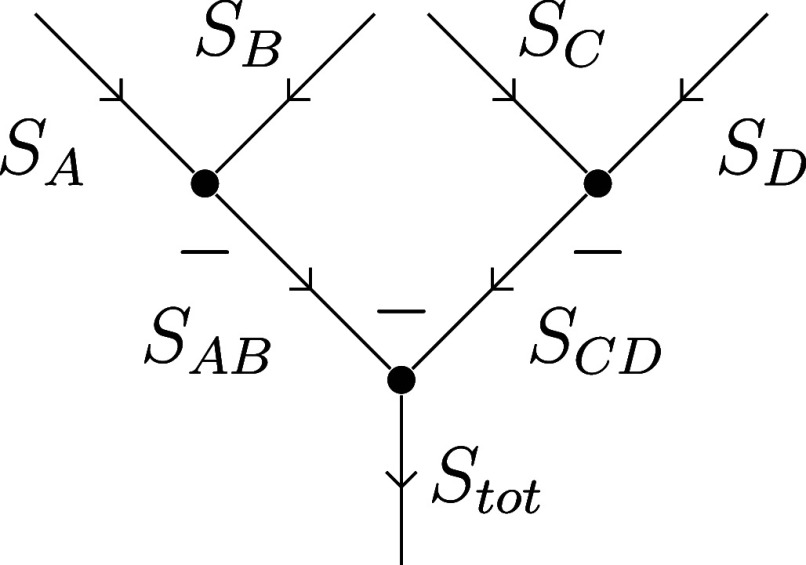
Coupling of four angular momenta in the ((AB)(CD)) scheme, closely resembling a binary tree.

Many transition-metal clusters like the oxygen evolving complex or FeMo cofactor are formed by four or more magnetic sites. The sequential coupling of local spins implied by eq 16 will suffice to construct the effective Hamiltonian for these compounds, but it is known that certain models admit diagonal form in nonsequential orderings. A relevant example is the four-site bilinear Heisenberg model:
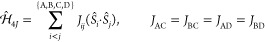
19which is diagonal in the ((AB)(CD)) coupling scheme.^77^ Drawn as a spin graph (see Figure 3), this arrangement of spins resembles a balanced binary tree, thus the abbreviation
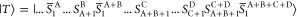
20will be used for these CSFs.

Local spins A, B, C, D are already defined for |*L*⟩ CSFs; hence, the remaining transformation from eq 16, (((AB)C)D) → ((AB)(CD)), can be expressed as a single Racah coefficient:
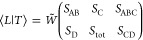
21allowing us to examine the properties of the ab initio wave function in this basis. As an example, an all-ferric [Fe_4_^(III)^S_4_(SMe)_4_] cubane model system will be considered.^33,78^ The singlet manifold of this particular cluster was previously studied with GUGA–FCIQMC.^33^ To illustrate the concept, we concentrate on the exactly diagonalizable *S*_tot_ = 8 block of the minimal CAS(20,20) comprising the Fe 3d orbitals. Calculations were performed with OpenMolcas,^57^ using the ANO–RCC basis set^62^ with contractions Fe(21s15p10d)/[5s4p2d], S(17s12p)/[4s3p], C(14s9p)/[2s1p], H(8s)/[1s] and the Cholesky decomposition of two-electron integrals with a threshold of 10^–4^.^63,64^

The model space of the *S*_tot_ = 8 manifold is spanned by six states, resulting from the coupling of four *S_i_* = . From the branching diagram, 170 open-shell CSFs can be identified in a localized basis, entirely similar to the left side of Figure 1. Table 5 lists the *L*_1_ norm, , and inverse participation ratio (IPR), , as measures of sparsity in this generic basis |*G*⟩ and compares it to the standard |*C*⟩, local sequential |*L*⟩, and binary tree |*T*⟩ coupling schemes in the site-ordered basis. Assuming the worst-case scenario, orbitals in |*G*⟩ are ordered such that electrons 1 and 2 are on different atoms, preventing partial fulfilment of Hund’s rule on the first site.

**Table 5:** Sparsity Measures for the Branching Diagram CSFs of the First Six Roots |*S*_tot_ = 8⟩ in the Singly Occupied Manifold |ψ̅⟩ of the CAS(20,20)^a^

*S*_tot_ = 8	| ψ̅_1_⟩	| ψ̅_2_⟩	| ψ̅_3_⟩	| ψ̅_4_⟩	| ψ̅_5_⟩	| ψ̅_6_⟩
***L*_1_ norm**						
|*G*⟩	10.4	10.3	10.3	11.4	7.86	10.4
|*C*⟩	6.80	6.76	6.70	5.82	6.80	6.45
|*L*⟩	1.46	1.97	1.96	1.72	1.46	1.69
|*T*⟩	1.07	1.48	1.48	1.06	1.06	1.04
**IPR**						
|*G*⟩	0.01	0.01	0.01	0.01	0.03	0.01
|*C*⟩	0.03	0.04	0.04	0.04	0.03	0.03
|*L*⟩	0.56	0.33	0.34	0.4	0.56	0.41
|*T*⟩	0.97	0.49	0.49	0.97	0.97	0.98

a Small values of the *L*_1_ norm and large IPRs indicate sparse wave functions.

Enforcing Hund’s rule on the first site with the standard representation |*C*⟩ reduces the number of appreciable CI coefficients from 170 to 50 and is accompanied by a significant increase in sparsity as quantified by the *L*_1_ criterion. The increase in IPR is much smaller, because strong mixing within this reduced space still is necessary to resolve Hund’s rule on sites B, C, and D. Proceeding to |*L*⟩ and |*T*⟩, only six nonvanishing CSFs remain and their squared coefficients are shown in Table 6.

**Table 6:** Weights of Wave Functions for the First Six *S*_tot_ = 8 Roots of the CAS(20,20) Projected onto the Heisenberg Manifold in the Local Sequential and Balanced Binary Tree Coupling Schemes^a^

*S*_tot_ = 8		|ψ̅_1_⟩	|ψ̅_2_⟩	|ψ̅_3_⟩	|ψ̅_4_⟩	|ψ̅_5_⟩	|ψ̅_6_⟩
Δ*E*/cm^–1^		0	14	16	76	76	157
**|*L*⟩ = |*S*_AB_, *S*_ABC_⟩**							
			0.48	0.50			
		0.30				0.68	
		0.68				0.30	
			0.07	0.07	0.44		0.41
			0.21	0.20	0.10		0.48
			0.23	0.22	0.45		0.09
**|*T*⟩ = |*S*_AB_, *S*_CD_⟩**							
	|3, 5⟩		0.48	0.50			
	|4, 4⟩	0.99					
	|4, 5⟩					0.99	
	|5, 3⟩		0.50	0.48			
	|5, 4⟩				0.99		
	|5, 5⟩						0.99

a Zeros were omitted to enhance legibility. Since roots 2 and 3 and roots 4 and 5 are (nearly) degenerate, mixing within the relevant subspaces is possible.

Although sparsity is maximized in |*T*⟩, as would be expected from eq 19, the largest gain is already realized by recoupling from |*C*⟩ to |*L*⟩, where Hund’s rule can be expressed on every site. This example makes a case in point for the |*L*⟩ basis, because the redox cycles in nature mostly feature metal centers of mixed-oxidation states for which |*T*⟩ CSFs are not expected to be more descriptive. Nevertheless, we presume that nonsequential couplings could be advantageous for partitioning large magnetic systems into smaller units whenever the subsystem exhibits a particular symmetry, e.g., treating the FeMo cofactor in terms of two magnetically coupled cuboids. Note that while eq 16 does not hold for inequivalent magnetic sites, the recoupling of CI vectors into local spin-adapted representations remains applicable. In these cases, a new recoupling diagram, similar to the example provided in Section 2, can be drawn and reduced to a sequence of Racah symbols and Kronecker deltas.

## 5 Conclusion

In this letter, we extended the effective Hamiltonian formalism to polynuclear transition metal complexes with local spins greater than one half by means of recoupling transformations. The method was used to assess a spin model for a [CaMn_3_^(IV)^O_4_] cubane with minimal and extended active space calculations. Enlarging the active space was shown to qualitatively change the magnetism from a three-parameter to a two-parameter interaction, illustrating the utility and importance of an exact mapping between ab initio and model wave functions. An extension to sparse solvers like DMRG or FCIQMC is readily possible, if CI coefficients can be efficiently extracted. We also discussed how chains of local spin-adapted functions can be transformed into nonsequential, treelike orderings to express additional permutational symmetries of model Hamiltonians in ab initio wave functions. Overall, the increased compactness of CI solutions in nonstandard coupling schemes portends a promising future for their use in sparse solvers like FCIQMC or as reference states for perturbation theory. It remains to be seen whether the additional sparsity compared to the standard basis outweighs the overhead of more complicated Hamiltonian matrix element evaluation.^79,80^

## Appendix A. Recapitulation of Angular Momentum Diagrams

Clebsch–Gordan coefficients, ⟨*j*_1_*m*_1_; *j*_2_*m*_2_|*j*_3_*m*_3_⟩, are the building blocks of the Racah–Wigner calculus. The angular momenta fulfill the triangle condition:

22

and sum to an integer . In graphical notation, they are represented by three lines connected to a semicircle vertex. Standard and contrastandard states are denoted by single and double arrows, respectively. SU(2) invariants are formed by summing over all magnetic quantum numbers of a standard/contrastandard product. Since bra/ket and ket/bra contractions are invariant under simultaneous time reversal, these contracted lines are not oriented, see also eqs (54) and (55) in ref (43). Note that coupling more than two angular momenta implies a summation over the magnetic quantum numbers of intermediate *j*, e.g.,

23

Although recoupling problems are conveniently expressed in terms of generalized Clebsch–Gordan coefficients, because of their cumbersome symmetries, it is convenient for the diagrammatic approach to use more symmetric 3–*jm* symbols instead. Following chapter 3.6 of ref (43), this conversion involves five steps (always referring to the initial diagram):^43^

(i) change the sign of the vertex,
(ii) keeping their direction, exchange all double arrows with single arrows,
(iii) add a factor  for the unique line, i.e., the line of bra or ket type appearing only once,
(iv) add a factor (−1)^2*j*^ for the first line encountered in going from the unique single (ket)/double (bra) arrow line into the direction indicated by the vertex sign (for a ket: *–* clockwise/*+* counter clockwise, for a bra: *–* counter clockwise/*+* clockwise),
(v) add a factor (−1)^2*j*^ for every time-reversed bra (outgoing double arrow).

For example:

24

Changing the node sign on a 3–*jm* symbol, corresponding to an odd permutation of columns, incurs a factor of (−1)^*j*_1_+*j*_2_+*j*_3_^. Unlike the bra/ket and ket/bra contractions of Clebsch–Gordan coefficients, the lines of contracted 3–*jm* symbols are oriented. Arrows on contracted lines can be inverted, introducing a factor of (−1)^2*j*^. A diagram with two external lines can be closed as follows:From a contracted graph, a sequence of line separations leads to irreducible 3*n*–*j* symbols. In the main text, we use the two- and three-line separation theorems:Closed and open boxes represent diagrams with fully and noncontracted lines, respectively.

The final expressions are written in terms of 3*j* and 6*j* symbols, the former of which is defined as

27

The 6*j* symbol is a contraction of four 3–*jm* symbols:

In standard form, all vertices have the same sign. The inner lines either all point toward or away from the center, whereas the outer arrows follow the direction indicated by the vertex sign. Inverting all signs and outer arrow directions is equivalent to reflecting the symbol on an outer axis. Arbitrary column permutations or the pairwise inversion of bottom and top elements leave the 6*j* symbol invariant, e.g.,

29

Many programming languages provide optimized libraries or wrappers to obtain the numerical values of 3*n*–*j* symbols.^81^

## Supporting Information Available

(A) RASSCF(27,24) orbital pictures, (B) absolute energies for the calculations in section 3, (C) model and effective Hamiltonians based on CASSCF/RASSCF for all spin manifolds of the [CaMn_3_(IV)O_4_] cubane, (D) explanation of extraction procedure for fragment wave function ansätze (PDF)

## Footnotes

1 In symmetric group parlance, defining *S*_B_ and *S*_C_ for the branching diagram CSFs is equivalent to reducing the representation Γ^*n*^ with respect to the subgroups π_*n*_A__, π_*n*_B__ and π_*n*_C__, *n*_A_ + *n*_*B*_ + *n*_C_ = *n*, instead of the canonical chain π_*n*–1_, ..., π_1_.^26^ By inspection of the corresponding Young tableaux one can verify that out of all nonvanishing CSFs in the right of Figure 1 only |*uuu uuu ddd*⟩ and |*uuu ddd uuu*⟩ have this property, i.e., the boxes labeled by (1,2,3), (4,5,6), and (7,8,9) form standard sub-tableaux.

2 Possible in OpenMolcas through a modification of the &RASSCF/FCIQMC interface.
