# Supplementary material for: Effective Hamiltonians from Spin-Adapted Configuration Interaction
Source: J Chem Theory Comput. 2025 Jan 8;21(2):539–48. doi: 10.1021/acs.jctc.4c01380 (PMC11780745; doi:10.1021/acs.jctc.4c01380)
Supplement: Supplementary file 1 — ct4c01380_si_001.pdf [file ct4c01380_si_001.pdf]

Supporting Information: Effective Hamiltonians from Spin–Adapted Configuration Interaction

Arta A. Safari* and Nikolay A. Bogdanov*

Max-Planck-Institute for Solid State Research, 70569 Stuttgart, Germany

E-mail: a.safari@fkf.mpg.de; n.bogdanov@fkf.mpg.de

Contents

S1 RASSCF Orbitals for the [$CaMn_3{}^{(IV)}O_4$] Cubane 2

S2 Absolute Energies for the [$CaMn_3{}^{(IV)}O_4$] Cubane 3

S3 Analytical Matrix for the 3J–3K Heisenberg Model 4

S4 Effective Hamiltonian from CASSCF(9,9) 5

S5 Effective Hamiltonian from RASSCF(27, 2, 2; 9, 9, 6) 6

S6 Effective Hamiltonians with Localised Active Space State Interaction 7

References 7

S1 RASSCF Orbitals for the [$CaMn_3^{(IV)}O_4$] Cubane

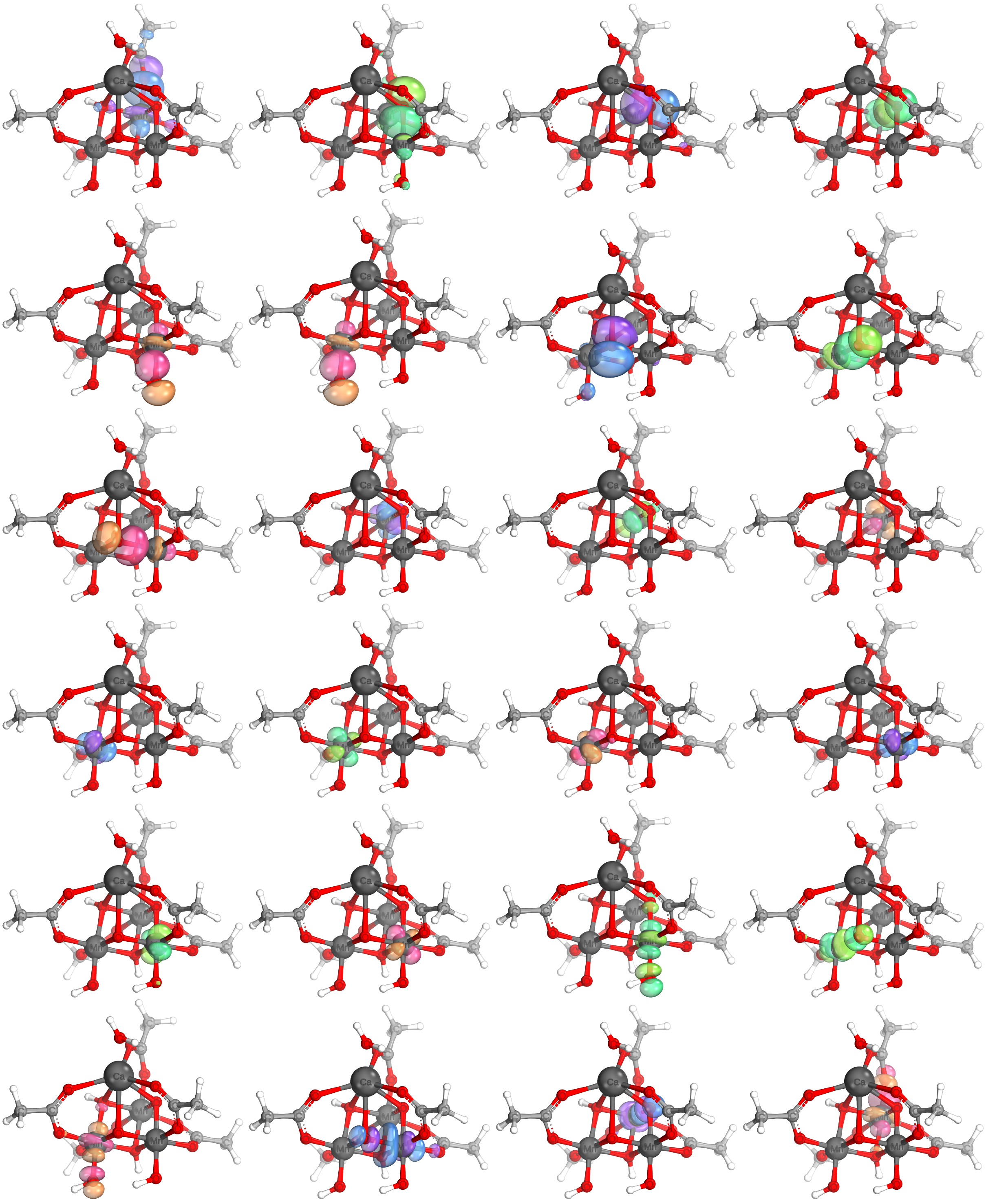

Figure S1: Split localised RASSCF(27, 2, 2; 9, 9, 6) orbitals used in the effective Hamiltonian mapping procedure.

S2 Absolute Energies for the [$CaMn_3^{(IV)}O_4$] Cubane

Table S1: Total energies in Hartree obtained at various levels of theory for the protonated [$CaMn_3^{(IV)}O_4$] cubane discussed in the main text. CI roots with the same total spin are listed in descending order.

	$S_{tot} = \frac{1}{2}$	$S_{tot} = \frac{3}{2}$	$S_{tot} = \frac{5}{2}$	$S_{tot} = \frac{7}{2}$	$S_{tot} = \frac{9}{2}$
CASSCF(9, 9)					
	-5815.75979963	-5815.76008349	-5815.76025078	-5815.76046090	-5815.76070581
	-5815.75963047	-5815.75992670	-5815.76005771	-5815.76020897	
		-5815.75978378	-5815.75982753		
		-5815.75955832			
RASSCF(27, 24)					
	-5816.01113429	-5816.01141063	-5816.01145458	-5816.01151340	-5816.01158419
	-5816.01096566	-5816.01115954	-5816.01120145	-5816.01125977	
		-5816.01098828	-5816.01103209		
		-5816.01090401			
+ftPBE					
	-5828.07716873	-5828.07722610	-5828.07700641	-5828.07669678	-5828.07628050
	-5828.07705221	-5828.07703292	-5828.07681467	-5828.07649991	
		-5828.07688971	-5828.07669272		
		-5828.07687372			
+ftBLYP					
	-5830.61378722	-5830.61383019	-5830.61358662	-5830.61324374	-5830.61278377
	-5830.61367154	-5830.61363695	-5830.61339524	-5830.61304674	
		-5830.61349280	-5830.61327368		
		-5830.61348064			
+ftSCAN_E0					
	-5441.50160947	-5441.50165847	-5441.50105524	-5441.50020835	-5441.49908071
	-5441.50135278	-5441.50123951	-5441.50064201	-5441.49978106	
		-5441.50092332	-5441.50037160		
		-5441.50089299			

S3 Analytical Matrix for the 3J–3K Heisenberg Model

$$\hat{\mathcal{H}}_{3J3K} = \sum_{i<j}^{\{A,B,C\}} J_{ij}(\hat{S}_i \cdot \hat{S}_j) + K_{ij}(\hat{S}_i \cdot \hat{S}_j)^2. \tag{S1}$$

Analytical matrix blocked by total spin with column labels $|S_{\text{tot}}, S_{AB}\rangle$. To enhance legibility, the substitutions $J_{3m} = J_{13} - J_{23}$, $J_{3p} = J_{13} + J_{23}$ and analogously for K_{3m} and K_{3p} were performed.

$\hat{\mathcal{H}}_{3J3K}$	$\lvert\frac{1}{2},1\rangle$	$\lvert\frac{1}{2},2\rangle$
$\langle\frac{1}{2},1\rvert$	$\frac{1}{16}(-44J_{12} - 20J_{3p} - 121K_{12} - 37K_{3p})$	$-\frac{1}{4}\sqrt{3}(2J_{3m} + 7K_{3m})$
$\langle\frac{1}{2},2\rvert$	$-\frac{1}{4}\sqrt{3}(2J_{3m} + 7K_{3m})$	$-\frac{3}{16}(4J_{12} + 12J_{3p} + 3K_{12} + 31K_{3p})$

$\hat{\mathcal{H}}_{3J3K}$	$\lvert\frac{3}{2},0\rangle$	$\lvert\frac{3}{2},1\rangle$
$\langle\frac{3}{2},0\rvert$	$-\frac{15}{16}(4J_{12} + 5(3K_{12} + K_{3p}))$	$-\frac{5}{8}\sqrt{3}(2J_{3m} + K_{3m})$
$\langle\frac{3}{2},1\rvert$	$-\frac{5}{8}\sqrt{3}(2J_{3m} + K_{3m})$	$\frac{1}{80}(-5(44J_{12} + 8J_{3p} + 121K_{12}) - 587K_{3p})$
$\langle\frac{3}{2},2\rvert$	$-\frac{3\sqrt{5}K_{3p}}{2}$	$-2\sqrt{\frac{3}{5}}(J_{3m} + 2K_{3m})$
$\langle\frac{3}{2},3\rvert$	0	$-\frac{3\sqrt{21}K_{3p}}{10}$

$\hat{\mathcal{H}}_{3J3K}$	$\lvert\frac{3}{2},2\rangle$	$\lvert\frac{3}{2},3\rangle$
$\langle\frac{3}{2},0\rvert$	$-\frac{3\sqrt{5}K_{3p}}{2}$	0
$\langle\frac{3}{2},1\rvert$	$-2\sqrt{\frac{3}{5}}(J_{3m} + 2K_{3m})$	$-\frac{3\sqrt{21}K_{3p}}{10}$
$\langle\frac{3}{2},2\rvert$	$-\frac{3}{16}(4J_{12} + 8J_{3p} + 3K_{12} + 29K_{3p})$	$-\frac{3}{8}\sqrt{\frac{7}{5}}(2J_{3m} + 9K_{3m})$
$\langle\frac{3}{2},3\rvert$	$-\frac{3}{8}\sqrt{\frac{7}{5}}(2J_{3m} + 9K_{3m})$	$\frac{9J_{12}}{4} - 3J_{3p} - \frac{27}{80}(15K_{12} + 29K_{3p})$

$\hat{\mathcal{H}}_{3J3K}$	$\lvert\frac{5}{2},1\rangle$	$\lvert\frac{5}{2},2\rangle$
$\langle\frac{5}{2},1\rvert$	$\frac{1}{80}(-220J_{12} + 60J_{3p} - 605K_{12} - 297K_{3p})$	$\frac{3}{4}\sqrt{\frac{7}{5}}(K_{3m} - 2J_{3m})$
$\langle\frac{5}{2},2\rvert$	$\frac{3}{4}\sqrt{\frac{7}{5}}(K_{3m} - 2J_{3m})$	$\frac{1}{16}(-12J_{12} - 4J_{3p} - 9K_{12} - 77K_{3p})$
$\langle\frac{5}{2},3\rvert$	$-\frac{3\sqrt{14}K_{3p}}{5}$	$-2\sqrt{\frac{2}{5}}(J_{3m} + 2K_{3m})$

$\hat{\mathcal{H}}_{3J3K}$	$\lvert\frac{5}{2},3\rangle$
$\langle\frac{5}{2},1\rvert$	$-\frac{3\sqrt{14}K_{3p}}{5}$
$\langle\frac{5}{2},2\rvert$	$-2\sqrt{\frac{2}{5}}(J_{3m} + 2K_{3m})$
$\langle\frac{5}{2},3\rvert$	$\frac{1}{80}(180J_{12} - 140J_{3p} - 405K_{12} - 373K_{3p})$

$\hat{\mathcal{H}}_{3J3K}$	$\lvert\frac{7}{2},2\rangle$	$\lvert\frac{7}{2},3\rangle$
$\langle\frac{7}{2},2\rvert$	$-\frac{3}{16}(4J_{12} - 8J_{3p} + 3(K_{12} + 7K_{3p}))$	$\frac{3}{8}\sqrt{3}(3K_{3m} - 2J_{3m})$
$\langle\frac{7}{2},3\rvert$	$\frac{3}{8}\sqrt{3}(3K_{3m} - 2J_{3m})$	$\frac{9}{16}(4J_{12} - 3(3K_{12} + K_{3p}))$

$\hat{\mathcal{H}}_{3J3K}$	$\lvert\frac{9}{2},3\rangle$
$\langle\frac{9}{2},3\rvert$	$\frac{9}{16}(4J_{12} + 4J_{3p} - 9(K_{12} + K_{3p}))$

S4 Effective Hamiltonian from CASSCF(9,9)

Left column: effective Hamiltonian obtained from CASSCF(9,9), right column: model Hamiltonian (S1) with CASSCF(9,9) parameters from Table 4 plugged in. Both blocked by total spin with column labels $|S_{\text{tot}}, S_{AB}\rangle$. All values are given in units of cm^{-1} relative to $|\frac{9}{2}, 3\rangle$.

$\hat{\mathcal{H}}_{\text{eff}}^{\text{CASSCF}}$	$\lvert\frac{1}{2},1\rangle$	$\lvert\frac{1}{2},2\rangle$
$\langle\frac{1}{2},1\rvert$	200	7
$\langle\frac{1}{2},2\rvert$	7	235

$\hat{\mathcal{H}}_{3J3K}^{\text{CASSCF}}$	$\lvert\frac{1}{2},1\rangle$	$\lvert\frac{1}{2},2\rangle$
$\langle\frac{1}{2},1\rvert$	200	7
$\langle\frac{1}{2},2\rvert$	7	235

$\hat{\mathcal{H}}_{\text{eff}}^{\text{CASSCF}}$	$\lvert\frac{3}{2},0\rangle$	$\lvert\frac{3}{2},1\rangle$	$\lvert\frac{3}{2},2\rangle$	$\lvert\frac{3}{2},3\rangle$
$\langle\frac{3}{2},0\rvert$	147	17		
$\langle\frac{3}{2},1\rvert$	17	165	12	
$\langle\frac{3}{2},2\rvert$		12	199	7
$\langle\frac{3}{2},3\rvert$			7	251

$\hat{\mathcal{H}}_{3J3K}^{\text{CASSCF}}$	$\lvert\frac{3}{2},0\rangle$	$\lvert\frac{3}{2},1\rangle$	$\lvert\frac{3}{2},2\rangle$	$\lvert\frac{3}{2},3\rangle$
$\langle\frac{3}{2},0\rvert$	147	17		
$\langle\frac{3}{2},1\rvert$	17	165	12	
$\langle\frac{3}{2},2\rvert$		12	199	7
$\langle\frac{3}{2},3\rvert$			7	251

$\hat{\mathcal{H}}_{\text{eff}}^{\text{CASSCF}}$	$\lvert\frac{5}{2},1\rangle$	$\lvert\frac{5}{2},2\rangle$	$\lvert\frac{5}{2},3\rangle$
$\langle\frac{5}{2},1\rvert$	105	14	
$\langle\frac{5}{2},2\rvert$	14	139	10
$\langle\frac{5}{2},3\rvert$		10	191

$\hat{\mathcal{H}}_{3J3K}^{\text{CASSCF}}$	$\lvert\frac{5}{2},1\rangle$	$\lvert\frac{5}{2},2\rangle$	$\lvert\frac{5}{2},3\rangle$
$\langle\frac{5}{2},1\rvert$	105	14	
$\langle\frac{5}{2},2\rvert$	14	139	10
$\langle\frac{5}{2},3\rvert$		10	191

$\hat{\mathcal{H}}_{\text{eff}}^{\text{CASSCF}}$	$\lvert\frac{7}{2},2\rangle$	$\lvert\frac{7}{2},3\rangle$
$\langle\frac{7}{2},2\rvert$	56	10
$\langle\frac{7}{2},3\rvert$	10	107

$\hat{\mathcal{H}}_{3J3K}^{\text{CASSCF}}$	$\lvert\frac{7}{2},2\rangle$	$\lvert\frac{7}{2},3\rangle$
$\langle\frac{7}{2},2\rvert$	56	10
$\langle\frac{7}{2},3\rvert$	10	107

S5 Effective Hamiltonian from RASSCF(27, 2, 2; 9, 9, 6)

Left column: effective Hamiltonian obtained from RASSCF(27, 2, 2; 9, 9, 6), right column: model Hamiltonian (S1) with RASSCF(27, 2, 2; 9, 9, 6) parameters from Table 4 plugged in. Both blocked by total spin with column labels $|S_{\text{tot}}, S_{AB}\rangle$. All values are given in units of cm^{-1} relative to $|\frac{9}{2}, 3\rangle$.

$\hat{\mathcal{H}}_{\text{eff}}^{\text{RASSCF}}$	$\|\frac{1}{2},1\rangle$	$\|\frac{1}{2},2\rangle$
$\langle\frac{1}{2},1\|$	108	16
$\langle\frac{1}{2},2\|$	16	127

$\hat{\mathcal{H}}_{3J3K}^{\text{RASSCF}}$	$\|\frac{1}{2},1\rangle$	$\|\frac{1}{2},2\rangle$
$\langle\frac{1}{2},1\|$	108	16
$\langle\frac{1}{2},2\|$	16	127

$\hat{\mathcal{H}}_{\text{eff}}^{\text{RASSCF}}$	$\|\frac{3}{2},0\rangle$	$\|\frac{3}{2},1\rangle$	$\|\frac{3}{2},2\rangle$	$\|\frac{3}{2},3\rangle$
$\langle\frac{3}{2},0\|$	79	40		
$\langle\frac{3}{2},1\|$	40	89	28	
$\langle\frac{3}{2},2\|$		28	107	16
$\langle\frac{3}{2},3\|$			16	136

$\hat{\mathcal{H}}_{3J3K}^{\text{RASSCF}}$	$\|\frac{3}{2},0\rangle$	$\|\frac{3}{2},1\rangle$	$\|\frac{3}{2},2\rangle$	$\|\frac{3}{2},3\rangle$
$\langle\frac{3}{2},0\|$	79	40	1	
$\langle\frac{3}{2},1\|$	40	89	28	
$\langle\frac{3}{2},2\|$	1	28	108	16
$\langle\frac{3}{2},3\|$			16	137

$\hat{\mathcal{H}}_{\text{eff}}^{\text{RASSCF}}$	$\|\frac{5}{2},1\rangle$	$\|\frac{5}{2},2\rangle$	$\|\frac{5}{2},3\rangle$
$\langle\frac{5}{2},1\|$	56	33	
$\langle\frac{5}{2},2\|$	33	75	23
$\langle\frac{5}{2},3\|$		23	103

$\hat{\mathcal{H}}_{3J3K}^{\text{RASSCF}}$	$\|\frac{5}{2},1\rangle$	$\|\frac{5}{2},2\rangle$	$\|\frac{5}{2},3\rangle$
$\langle\frac{5}{2},1\|$	56	33	
$\langle\frac{5}{2},2\|$	33	75	23
$\langle\frac{5}{2},3\|$		23	103

$\hat{\mathcal{H}}_{\text{eff}}^{\text{RASSCF}}$	$\|\frac{7}{2},2\rangle$	$\|\frac{7}{2},3\rangle$
$\langle\frac{7}{2},2\|$	30	24
$\langle\frac{7}{2},3\|$	24	57

$\hat{\mathcal{H}}_{3J3K}^{\text{RASSCF}}$	$\|\frac{7}{2},2\rangle$	$\|\frac{7}{2},3\rangle$
$\langle\frac{7}{2},2\|$	30	24
$\langle\frac{7}{2},3\|$	24	58

S6 Effective Hamiltonians with Localised Active Space State Interaction

In the localised active space (LAS) framework, the wave function is expressed as an anti–symmetrised tensor product of fragment wave functions $|n_i\rangle$:

$$|\text{LAS}\rangle = \bigwedge_{i}^{\text{fragments}} |n_i\rangle \,, \tag{S2}$$

where it was assumed that the doubly occupied orbitals are frozen. The $|n_i\rangle$ are fragment wave functions with well–defined subsystem quantum numbers. Missing inter-space correlation is obtained by forming linear combinations of LAS–states in LAS state interaction (LASSI). Recently, a semi–automatic procedure[1] for LASSI was introduced that operates with the number of inter–fragment excitations r and intra–fragment excited states q, denoted LASSI[r, q]. In the limit $r, q \to \infty$ the method is equivalent to a CAS ansatz.

For active spaces comprising multiple magnetic sites, equation (S2) closely resembles the uncoupled basis of a spin Hamiltonian $|j_1 m_1;\ j_2 m_2;\ \ldots;\ j_i m_i\rangle$ where the conserved subsystem quantum numbers on site i are j_i and m_i. Since $m_{\text{total}} = \sum_i m_i$ is a good quantum number for the models we consider, the Hamiltonian in the uncoupled basis is blocked by m_{total}. Choosing the sub–block with the smallest m_{total} provides the smallest non–redundant form of the Hamiltonian that can be used to obtain the coupling parameters. If each $|n_i\rangle$ corresponds to a magnetic site, LASSI[0, 1] recovers up to a phase the Clebsch–Gordan coefficients occurring in the tensor product space reduction of $|j_1 m_1;\ j_2 m_2;\ \ldots;\ j_i m_i\rangle$. Consequently, rather than using the recoupling procedure presented in the main manuscript, general LASSI[r, q] states can be projected onto the LASSI[0, 1] manifold to form the effective Hamiltonian.

References

(1) Agarawal, V.; King, D. S.; Hermes, M. R.; Gagliardi, L. Automatic State Interaction with Large Localized Active Spaces for Multimetallic Systems. *Journal of Chemical Theory and Computation* **2024**, *20*, 46544662, DOI: 10.1021/acs.jctc.4c00376.
